# Poniéndole la lupa a la estrategia farmacoinvasiva

**DOI:** 10.47487/apcyccv.v5i1.344

**Published:** 2024-03-19

**Authors:** Frank W. Britto

**Affiliations:** 1 Instituto Nacional Cardiovascular INCOR, Lima, Perú. Instituto Nacional Cardiovascular INCOR Lima Perú

*Sr. Editor:*

Deseo felicitar a los autores de la oportuna revisión titulada «Propuesta de manejo inicial del infarto de miocardio con elevación del segmento ST no complicado en centros sin capacidad de intervención coronaria percutánea en el Perú» ^1^. Insisto en lo oportuno del artículo, porque recientemente EsSalud ha aprobado la directiva para la Red de Atención del Infarto Agudo de Miocardio (RADIAM) ^2^.

La guía de práctica clínica de EsSalud para el manejo del infarto agudo de miocardio ^3^, la directiva RADIAM y el presente artículo, señalan la necesidad imperiosa de transferir a los pacientes con infarto de miocardio con elevación del segmento ST (IAMCEST), luego de fibrinolisis exitosa y estables, a centros de referencia con capacidad de intervención coronaria percutánea. Esta estrategia, llamada «farmacoinvasiva» ha demostrado la disminución del riesgo de reinfarto, desarrollo de isquemia recurrente y de falla cardiaca ^4^.

Como señalan los autores, luego de dos horas de la fibrinolisis y antes de las 24 h del inicio del dolor, se requiere transferir al paciente para realizar una coronariografía y realizar una angioplastia de la arteria responsable del infarto, si está indicada.

Pero, ¿qué conducta tomar si el centro que realiza la fibrinolisis no puede transferir al paciente dentro de las 24 h?, ya sea por razones geográficas (lejanía, falta de medios de transporte con regularidad) o por razones logísticas (presupuesto, lentitud administrativa, fines de semana). Los autores, con buen criterio, recomiendan a los centros sin capacidad de intervención coronaria percutánea, realizar un «mapeo» de los tiempos de transferencia. Pero, hecho esto, ¿qué recomendamos?; ¿existen datos que sustenten la trasferencia, después de las 24 h?; si decidimos transferir, ¿lo hacemos con todos los pacientes?

La guía de práctica clínica canadiense no tiene un nivel de evidencia para este escenario, pero recomienda que, en este caso, solo se trasfieran los pacientes de alto riesgo ^4^; en tanto que la guía de práctica clínica argentina para la reperfusión del infarto agudo de miocardio recomienda únicamente trasferir, en las primeras 24 h, a los pacientes de alto riesgo, además señala que el manejo conservador de aquellos sin alto riesgo es seguro ^5^.

Por último, la guía de práctica clínica AHA/ACC para la revascularización miocárdica, coloca esta conducta como IIa B-A ^6^, mientras que la NICE no especifica un tiempo límite, recomendándola durante la hospitalización ^7^; a diferencia de la reciente guía de práctica clínica europea para el manejo de los síndromes coronarios agudos, que la coloca como IA ^8^, aunque todas las guías comparten las mismas referencias bibliográficas.
